# Mixed-methods analysis of personal growth in an equity-centered leadership development program

**DOI:** 10.1017/cts.2024.597

**Published:** 2024-11-08

**Authors:** Josephine McKelvy, Chenguang Du, Michelle Song, Tara Carr, Rachel Berthiaume, Giselle Corbie, Claudia Fernandez, Gaurav Dave

**Affiliations:** 1 Abacus Evaluation, UNC School of Medicine, Department of Social Medicine, University of North Carolina at Chapel Hill, Chapel Hill, NC, USA; 2 School of Psychology, Northwest Normal University, Lanzhou, China; 3 UNC Gillings School of Global Public Health, Department of Maternal and Child Health, University of North Carolina at Chapel Hill, Chapel Hill, NC, USA; 4 Center for Health Equity Research, UNC School of Medicine, Department of Social Medicine, University of North Carolina at Chapel Hill, Chapel Hill, NC, USA

**Keywords:** Equity-centered leadership training, Clinical Scholars, self-efficacy, mixed methods, confirmatory factor analysis, thematic analysis, program evaluation

## Abstract

**Introduction:**

Self-efficacy (or the belief in one’s ability to effect change) often moderates the relationship between education, interest, and actions in evaluations of training programs that prepare community-based investigators in the clinical and translational sciences workforce. Such evaluations, however, tend to emphasize individual-level attitudes when there are also community- or organizational-level outcomes impacted.

**Methods:**

This study uses a novel sequential, explanatory mixed-methods design to explore multiple levels of self-efficacy (or self-awareness of personal growth in leadership) in the Clinical Scholars program, an equity-centered leadership development program for mid- to later-career healthcare professionals. Our design involves: (1) bivariate correlations and confirmatory factor analysis of self-assessed competencies across all program participants to identify emergent combinations of competencies, which informed (2) more nuanced thematic coding of participants’ stories of most significant change in their personal and professional lives, as a result of the program.

**Results:**

In unpacking their accounts of personal leadership styles (that aligned with our quantitative analyses of competencies), we found that participants demonstrated multiple competencies simultaneously. Specifically, they employed emotionally intelligent learning and consensus-building dialogue to manage conflict for interpersonal impact. Additionally, they used this combination of skills to unite diverse stakeholders under a shared vision in order to lead and manage organizational change where all colleagues’ contributions were valued.

**Conclusion:**

Together, these methods extend our understanding of personal growth in leadership as an outcome of the program in terms of individual- and organizational-level impacts, using representative quantitative self-assessments to categorize rich qualitative descriptions.

## Introduction

In evaluating workforce training programs to prepare community-based investigators in the translational science workforce, self-efficacy (or the belief in one’s ability to effect change) moderates the relationship between the intervention and outcomes in career interest and actions [1–3]. Such assessments, however, tend to emphasize individual-level attitudes when subsequent actions, resulting from those beliefs, can also reveal broader impacts of the program. This study offers a novel method to investigate a specific form of efficacy: self-awareness of personal growth in leadership among participants, as an outcome of the Clinical Scholars program, with individual- and organizational-level impacts. We define this awareness or belief as the understanding of one’s personal leadership style within systems of relationships, environments, and institutions that inhibit or enable health equity (i.e., the ideal conditions where “everyone has a fair and just opportunity to be as healthy as possible”) [4,5]. But what skills, abilities, and attitudes make up participants’ leadership styles?

This paper provides an innovative, mixed-method approach to evaluating personal growth in five cohorts of participants in the Clinical Scholars program: a three-year, equity-centered leadership development program for mid- to later-career healthcare professionals. The cohorts comprise teams of licensed clinical providers or administrators in work sectors such as colleges and universities; hospitals, federally qualifying health centers, and outpatient care; community-based organizations, non-profits, and government entities; preK-12 schools; and veterinary services across the country. Participants apply as multidisciplinary teams to solve a “wicked problem” (that impacts global health across sectors) [4] through a focused project, and each team receives a yearly stipend of $35,000 for this work.

This program facilitates skill growth in cross-sector collaboration to address root causes of health inequities by using a learning and doing model. In this model, participants engage in a robust synchronous intensive (either onsite or virtually) and distance-based curriculum (both synchronous and asynchronous, both group and self-directed). Program faculty, invited presenters, and facilitators led courses and mentored teams as they applied their learning through community-based projects. Clinical Scholars is overseen by Co-Directors, Drs. Giselle Corbie and Claudia Fernandez, and funded by the Robert Wood Johnson Foundation (RWJF), as a part of its Leadership for Better Health: Change Leadership Programs that were designed in response to its 2014 Culture of Health Action Framework [6]. The curriculum (developed by the Co-Directors; Deputy Directors, Melissa A. Green, Kathy Donnald, and Rachel Berthiaume; and consultants, Drs. Angela Rosenberg and Katie Brandert, and other curriculum thought partners from the academic, business, non-profit and community sectors) involves 25 competencies seen as critical to developing the nuanced and sophisticated leadership skills that best support the interdisciplinary work that today’s complex health challenges require [7].

The above program team and RWJF made multipronged recruitment efforts to solicit team-based applications for the competitive, multistage selection process. Both entities promoted the program via print and digital resources, in-person events, webinars, and word of mouth through networks of health professional organizations, health profession educational communities, and public and private healthcare systems, accepting applications annually from January to March via the RWJF website. The program team and the Advisory Committee screened, reviewed, and scored applications for select interviews, ultimately inviting 10% of applicant teams to be finalists per cohort.

Our contribution to innovations in evaluating clinical and translational science training involves using quantitative analyses to identify additional qualitative elements that compose self-awareness of personal leadership growth in participants’ stories of most significant change. First, we outline how previous single-method studies informed our sequential, explanatory mixed-methods design for this study [8] (i.e., using statistical analyses of competency assessments to expand our thematic coding of qualitative change stories). Next, we present the quantitative findings from confirmatory factor analyses (CFA), resulting in six additional competencies that were highly related to the primary competency of interest: self-awareness. These competencies informed our focused qualitative analysis. Then we illustrate the emergent combinations of competencies (which compose multiple facets of participants’ self-awareness of personal leadership growth) using direct quotes from participants’ change stories. Finally, we discuss how this mixed-method approach triangulated findings to expand our understanding of a latent construct, like self-awareness. Together, these methods extend our understanding of personal growth in leadership as an outcome of the Clinical Scholars program in terms of individual-, interpersonal-, and organizational-level impacts, using representative quantitative self-assessments to categorize rich qualitative descriptions.

## Materials and methods

This mixed-method study explores the thematic construct of personal growth in leadership, based on the competency that showed the most quantitative growth over time: self-awareness. We use a sequential, explanatory mixed-methods design, which involves quantitative analyses of self-assessed competencies throughout the three-year program, followed by qualitative analyses of most significant change stories to contextualize those representative findings in terms of impactful changes in participants’ lives after the Clinical Scholars program [8–10].

In a previous longitudinal analysis of participants’ 25 competencies in the Competency Evaluation Survey [11], the dimension of self-efficacy showed the most growth but also fluctuated over time, decreasing between Months 6 and 18 (midpoint) before plateauing at Month 36 (endpoint) without further context. In another prior thematic analysis of Cohort 1’s change stories [12], the core competency of self-awareness constituted a significant outcome of the program and shaped the codebook for analyzing qualitative change stories. Applying this a priori codebook to subsequent cohorts’ stories, however, mostly revealed short statements (rather than impactful stories) that saturated around career advancement throughout the COVID-19 pandemic. To triangulate and extend these findings, we illustrate the primary variable, self-awareness, using factor analysis of data collected from a representative evaluation survey of all program participants to inform our thematic analysis of qualitative most significant change (MSC) stories across four of the five total cohorts.

**Figure 1.**
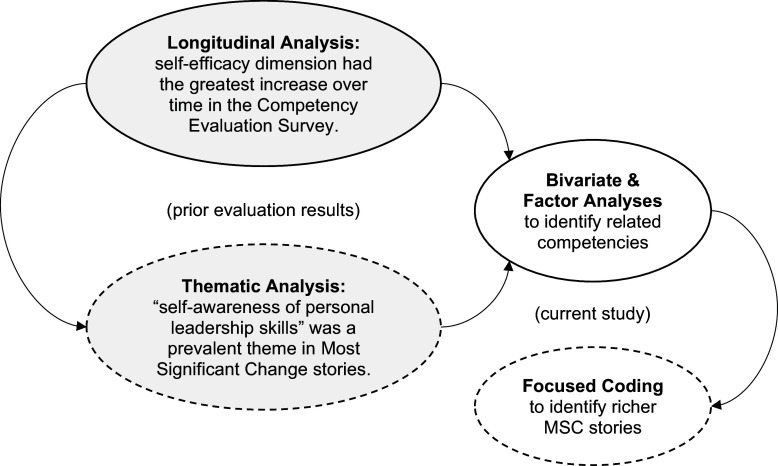
Sequence of quantitative and qualitative methods in prior evaluation results and this study.

Specifically, we use bivariate correlations and CFA to identify additional thematic constructs for a more nuanced qualitative analysis. The emergent combinations of these competencies (highly related to self-awareness) reveal attributes of personal growth in leadership that are greater than the sum of its parts. See Figure 1 for a diagram of how these multiple methods in prior published results (in gray circles) informed this current study (in white circles).

Together, the quantitative methods (in the solid circles) and qualitative methods (in the dashed circles) expand our understanding of personal growth in leadership (not only at an individual level but also interpersonally and organization- and community-wide) as an outcome of the Clinical Scholars program [9,10].

### Instruments, sample, and triangulation from prior evaluation results

The evaluation team used the Kirkpatrick model [13–15] to develop the Clinical Scholars Competency Evaluation Survey and collected program participants’ perceptions of their skills at multiple time points, using email links in Research Electronic Data Capture (REDCap). Participants used seven-point Likert scale items (1 = low to 7 = high) to self-assess four dimensions of learning (i.e., self-efficacy; attitude; knowledge; intent to use) for each of the 25 competencies that fell within four leadership domains (i.e., personal leadership; interpersonal skills; organizational impact; community and systems impact), which are based on the levels of the socioecological model.

Of the 173 finalists (across all five cohorts) who were eligible to enroll in the Clinical Scholars program, 162 Fellows participated and completed the program. The evaluation team collected demographic data from all 173 eligible finalists to convey the diversity of the potential cohorts. We then collected competency data from the 162 program participants via the evaluation survey across four time points when skill development, knowledge, and learning took place (i.e., Months 0 or baseline, 6, 18 or midpoint, and 36 or when participants graduated from the program). In this article, we present the latent construct that emerged from the representative survey data from these 162 participants and illustrate that construct with the thematic analysis of voluntary change stories. Because we are examining competencies related to self-awareness – and not individual-level change in self-ratings over time – we only excluded participants with missing information on all time points from our analysis, in order to reduce listwise deletions of missing ratings. (See Table 1 in the Supplementary Materials for more information about the survey constructs.) The University of North Carolina at Chapel Hill Institutional Review Board approved all evaluation research activities (Study #16-1817). Table 1 lists the sample characteristics of these participants, the majority of which identified as white and women. Almost half were mid-career, and almost one-third were physicians.

**Table 1.** Finalists’ demographics & participants’ response rates

Demographic	Count*	(%)*	Survey Respondents**	Change Stories Collected**
Cohort				
1 (2016–2019)	32	(18.50%)	29/29 (100.00%)	26/29 (89.66%)
2 (2017–2020)	35	(20.23%)	33/33 (100.00%)	14/33 (42.42%)
3 (2018–2021)	35	(20.23%)	31/31 (100.00%)	15/31 (48.39%)
4 (2019–2022)	35	(20.23%)	35/35 (100.00%)	15/35 (42.86%)
5 (2020–2023)	36	(20.81%)	29/34 (85.29%)	0/34 (0.00%)
Gender				
Women	126	(72.83%)		
Men	41	(23.70%)		
Unreported	6	(3.47%)		
Race				
White	104	(60.12%)		
Black/African American	25	(14.45%)		
Asian	20	(11.56%)		
Multi-Racial	7	(4.05%)		
Native Hawaiian or Pacific Islander	4	(2.31%)		
American Indian/Alaska Native	2	(1.16%)		
Other or Unreported	11	(6.36%)		
Discipline				
Physician	56	(32.37%)		
Nurse/Nurse Practitioner/Physician Asst.	33	(19.08%)		
Social Worker/Clinical Counselor	26	(15.03%)		
Psychologist	18	(10.40%)		
Pharmacist	8	(4.62%)		
Dentist	3	(1.73%)		
Dietician/Nutritionist	3	(1.73%)		
Occupational or Physical Therapist	4	(2.31%)		
Veterinarian	3	(1.73%)		
Speech-Language Pathologist	1	(0.58%)		
Complementary Medicine	1	(0.58%)		
Other or Unreported	17	(9.83%)		
Career Level				
Early career (0-5 years of clinical experience)	17	(9.83%)		
Mid-career (6-15 years of clinical experience)	81	(46.82%)		
Advanced (16+ years of clinical experience)	61	(35.26%)		
Unreported	14	(8.09%)		

* Counts and percentages of demographics derived from the applications of 173 finalists selected to enroll in the Clinical Scholars program.

** Percentages derived from the 162 participants who completed the program.

We computed a mean competency score for each of the 25 competencies at each time point by averaging the ratings of the four dimensions (i.e., self-efficacy; attitude; knowledge; use) for each participant. In 2017, the evaluation team changed the wording of a survey item on the dimension of use, in response to participant feedback for additional clarity. Because of this revision, we excluded Cohort 1’s competency assessments for Months 0 (baseline) and 6 from our analysis. Compared to attitude, knowledge, and use, the dimension of self-efficacy rated highest across all 25 competencies in the four domains [11].

Upon completing the program, participants also reflected on the most significant change they experienced in their personal and professional lives. The evaluation team emailed all participants an invitation to share written responses or video recordings in their final program reports. (See the Supplementary Materials for this prompt.) On average, about half of each cohort submitted change stories, apart from Cohort 5, who have not yet completed the program. The COVID-19 pandemic may have limited Cohorts 2 through 5 from engaging in the program and its evaluations, compared to Cohort 1, due to quarantine and increased responsibilities for patient care.

The evaluation team included all submitted stories verbatim (with the exception of identifying details) in the thematic analysis. We performed an independent *t*-test to compare the average competency scores at endpoint (Month 36, when participants shared their stories) between participants who submitted a change story and those who abstained. There was not a significant difference in average self-awareness scores between these groups at endpoint (*t*(104) = −1.33, *p* = 0.19) though those who submitted a change story (*M* = 6.11, SD = 0.55) tended to rate themselves slightly higher in self-awareness than those who did not submit a story (*M* = 5.96, SD = 0.70) upon completing the program. Competency scores range from 1 to 7. (See Table 2 in the Supplementary Materials for more information about the respondents’ and non-respondents’ average competency scores.)

**Table 2.** Correlation coefficients of self-awareness with eight competencies that had a large effect size across three or more time points

Competency		Month 0	Month 6	Month 18	Month 36	Frequency
Communication^a^	(COM)	0.697	0.754	0.663	0.747	4
Emotional Intelligence^a^	(EIN)	0.537	0.631	0.582	0.682	4
Conflict Management^a^	(CMG)	0.616	0.601	0.598	0.703	4
Leading Change/Change Management^b^	(LCM)	0.552	0.576	0.533	0.507	4
Commitment to Intercultural Development	(CDI)	0.560	0.625		0.607	3
Organizational Culture^b^	(IOC)	0.560	0.638		0.594	3
Visioning^b^	(VIS)	0.601	0.591		0.534	3
Social Justice	(SJU)	0.543	0.571		0.609	3

Only statistically significant coefficients (p ≤ 0.05) with large effect sizes (*r* ≥ 0.5) are listed.

a Top three competencies with statistically significant correlations with self-awareness and large effect sizes across four time points.

b Additional competencies with high loadings in CFA models.

We use a mixed-method approach to further explore the concept of self-awareness of personal leadership growth, as it relates to self-efficacy (or the belief in one’s ability to effect change), by triangulating the quantitative findings on the dimension of self-efficacy from the evaluation survey [11] and the competency of self-awareness in the change stories in previous studies. In these studies, self-efficacy showed the most growth over time (via representative surveys of the cohorts) and self-awareness emerged as a key theme (in the complementary change stories) [12]. We define our primary concept of self-awareness as demonstrating understanding of one’s personal leadership style in the context of organizations and systems of power.

### Qualitative thematic analysis

Using the qualitative analysis software, NVivo, the evaluation team inductively open coded Cohort 1’s change stories (using the participants’ words to label the topics emerging from the stories) as well as deductive codes (based on the 25 quantitatively assessed competencies) to develop a codebook of thematic constructs [16,17]. However, applying this codebook to subsequent cohorts’ stories led to findings that saturated mostly around participants’ career advancement. Again, given the extensive demands on healthcare providers’ time during the pandemic, it is not surprising that the latter cohorts focused more narrowly on their career experiences. Nevertheless, these limited results steered us toward quantitative analyses to identify additional combinations of thematic constructs in exploring how participants articulated the dimension of self-efficacy and the competency of self-awareness in their stories.

### Bivariate correlation and factor analyses

Using the statistical analysis software R (version 4.2.2), we first computed Pearson correlation coefficients between self-awareness and the other 24 competencies. Second, we selected competencies at each time point with statistically significant coefficients (*p* ≤ 0.05) [4]. Third, we kept competencies that had a large effect size (in at least three out of the four time points) for subsequent analyses (*r* ≥ 0.5). Fourth, we conducted a CFA for each time point to test our hypothesis that a unidimensional latent construct (or concept) exists among these observed competencies related to self-awareness – a construct with a known factor structure, based on prior findings [11,12]. We conducted logistic regression analyses to explore how demographic variables could predict the missing values from the competency scores and used full information maximum likelihood (FIML) estimation to handle the missing values in the current study.

### Sequential, explanatory mixed-methods design

After conducting these quantitative analyses, we returned to the change stories for more focused thematic coding; that is, identifying accounts that referenced related competencies with the top correlations and loadings from our statistical modeling rather than each of the 25 competencies individually. This round of coding sorted stories deductively by the operational definitions of select competencies (identified in the quantitative analyses) while also inductively defining emergent *combinations* of competencies that can illustrate latent factors related to the competency of self-awareness [16,17].

## Results

### Bivariate correlation and factor analyses

Table 2 shows the Pearson correlation coefficients between self-awareness and eight of the remaining 24 competencies that were statistically significant (*p* ≤ 0.05) with a large effect size (*r* ≥ 0.5) in at least three out of the four time points (i.e., results from steps 1–3 for our bivariate correlation analysis).

Some competencies were consistently and highly associated with self-awareness across all four time points. These include communication (COM), emotional intelligence (EIN), conflict management (CMG), and leading change/change management (LCM). The remaining competencies with large effect sizes across three time points include commitment to intercultural development (CDI), organizational culture (IOC), visioning (VIS), and social justice (SJU). Self-assessed competencies tended to increase between Months 0 (baseline) and 6 but then decreased between Months 6 and 18 (midpoint) before plateauing in Month 36 (end point). This dip at midpoint may explain why these competencies may not have had large effect sizes at Month 18.

**Figure 2.**
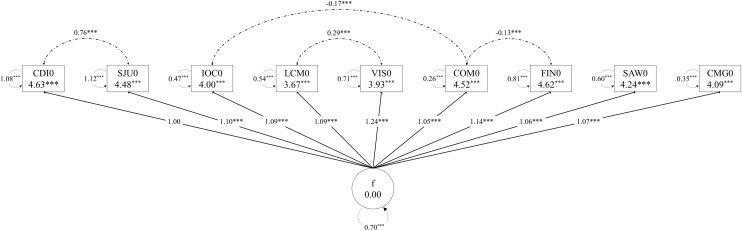
Confirmatory factor model for Month 0 with unstandardized loadings.

We conducted CFA with the nine competencies above (including self-awareness) across all four time points to confirm whether a single latent construct could explain the variance and covariance structure of these competencies and how stable this one-factor model could be. Figure 2 shows the CFA model for the baseline data, which maps the internal factor structure of the self-assessed competencies before Fellows participated in the program. (See Figures 1 to 3 in the Supplementary Materials for the CFA models from Months 6 to 36.)

Table 3 shows the top factor loadings for the CFAs that were significant (*p* ≤ 0.05). The range of the loadings (0.647–0.870) indicate that the variance explained by the common factor ranged from 41.86% to 75.69%.

**Table 3.** Factor loadings of the top competencies for the confirmatory factor analyses, in addition to the top three competencies highly correlated with self-awareness

Competency		Month 0	Month 6	Month 18	Month 36
Leading Change/Change Management ^b^	(LCM)	0.778	0.792	0.866	0.852
Organizational Culture ^b^	(IOC)	0.798	0.812	0.838	0.804
Conflict Management ^a^	(CMG)	0.833	0.800	0.725	0.860
Communication ^a^	(COM)	0.864	0.776	0.647	0.870
Visioning ^b^	(VIS)	0.776	0.753	0.760	0.748
Self-Awareness	(SAW)	0.750	0.782	0.632	0.821
Emotional Intelligence ^a^	(EIN)	0.725	0.732	0.651	0.720

Only the top loadings beyond the highly correlated competencies are listed.

a Top three competencies with statistically significant correlations with self-awareness and large effect sizes across four time points.

b Additional competencies with high loadings in CFA models.

To evaluate the model fit, we applied the following goodness-of-fit statistics: chi-square test, Tucker–Lewis index (TLI), comparative fit index (CFI), root mean square error of approximation (RMSEA), and standardized root mean residual (SRMR). According to Table 4, the CFA model reasonably fit the observed data in each time point as TLI and CFI are all above 0.90 (range: 0.961–0.982), and RMSEA and SRMR are all below 0.08 (range: 0.036–0.080) [18–20].

**Table 4.** Goodness-of-fit values

Time Point	*χ*^2^	df	*P*	TLI	CFI	RMSEA	SRMR
Month 0	36.220	23	0.039	0.968	0.980	0.074	0.037
Month 6	38.301	23	0.024	0.961	0.975	0.080	0.040
Month 18	30.938	20	0.056	0.968	0.982	0.066	0.037
Month 36	40.139	23	0.015	0.965	0.977	0.080	0.036

Having identified the top three competencies highly related to self-awareness (i.e., COM, EIN, and CMG) and three additional competencies with high factor loadings (i.e., LCM, IOC, and VIS), we then focus coded change stories, using the operational definitions of these six competencies [21]. We conducted a series of logistical regression models using gender, race, discipline, and career level to predict the missingness for each of the 9 variables used for CFA at each time point. The results indicate only IOC, EIN, and LCM at Months 0 and 6 could be significantly predicted by career level. This indicates the missing values of IOC, EIN, and LCM are more likely to be missing at random, while other variables may be missing completely at random. This helps validate the usage of FIML estimation to handle missing values.

### Qualitative thematic analysis

In the inductive open coding of change stories, self-awareness of personal leadership growth was the most prevalent theme, but the analysis mostly comprised single sentences about individual-level career advancement, possibly due to participants’ limited engagement in the program, due to increased patient care throughout the pandemic. This finding led to additional quantitative analyses to identify combinations of competencies that were highly related to the competency, self-awareness.

Conceptually, the operational definitions of highly correlated competencies (i.e., COM, EIN, and CMG) were linked when participants’ awareness of their strengths and weaknesses manifested in their stories as problem-solving dialogue in tense group interactions. One emergent property of this cluster of competencies (or resulting feature that is greater than the sum of its parts) includes facilitating consensus building while learning from others’ experiences.

Likewise, the additional competencies with high factor loadings over time in our factor analysis (i.e., LCM, IOC, and VIS) were conceptually connected as this combination of skills involved the process of leading or managing change in organizational culture through a shared mission (often in support of a vision focused on health equity) that created an inclusive and engaging work environment or community. Participants implicitly used empathetic consensus-building to realize a shared vision within groups.

The following change stories illustrate these emergent combinations of core competencies at multiple domains of personal, interpersonal, and organizational impacts of participants’ leadership development.

#### Emotional intelligence in communication and conflict management

Our bivariate correlations show that the top three competencies of communications, emotional intelligence, and conflict management were associated with self-awareness. In their stories, participants cited actions they took to understand others’ feelings and perspectives, in order to manage interpersonal conflict through communication. This combination of competencies reveals an emergent aspect of personal leadership growth (greater than the sum of its competencies) when participants empathized with and learned from others to create a more inclusive culture in their social networks and communities.

A participant from Cohort 4 listed the following communication skills – emphasizing both empathy and critical thinking – that they applied to their church, where such “crucial conversations” to move disagreement into action [22] were not yet happening:

“During my time as a Clinical Scholar, I learned to listen first to other people. We read a book entitled, Crucial Conversations, and participated in online learning about the topic. We practiced having these conversations with our Clinical Scholars Teams. We learned how to communicate in effective ways during times of crisis and conflict and controversy; how to communicate regret if we inadvertently offend someone; and how to communicate gratitude for what we learned from a difficult conversation.”

These skills paralleled those of other participants who used similar interpersonal skills in a variety of settings. For instance, a practitioner from Cohort 1 described a confrontation with a coworker over a new administrative protocol while they were treating a patient. Instead of internalizing the miscommunication as a personal failing, this participant expressed their appreciation for the coworker’s dedication, in addition to their own need to maintain collegiality in the exam room. This dialogue resulted in both colleagues taking accountability for their parts in the dispute. The participant reflected:

“I feel like the experience helped us bond. Approaching him in that way allowed us both to put our guards down. I’m not sure how I would have handled this in the past. I think I would probably not have been as confident in my approach, and I would not have understood that there are always unseen contributing factors in any interaction between two people or even two organizations. The Clinical Scholars program has taught me to always consider what I don’t know from the other person’s viewpoint and to ask questions with the intent of listening rather than responding.”

This participant contemplated how their emotional reactions to conflict have changed because of the program and how they intend to inquire more, actively listen, and gain others’ perspectives to better understand the conditions of tense situations.

A participant from Cohort 3 also conveyed how they harnessed emotions and relationships in their values and beliefs about leadership:

“The most significant addition is my change in leadership philosophy that includes leading by listening, encouraging, and empowering. As a trauma informed leader, I can now relate to those who suffer with greater empathy, respect, and passion. Leading from the heart, instead of the mind, yields tangible, durable, and flexible solutions to the challenges of today’s complex environments. I’m a more present, focused thinker, capable to gaining consensus and able to have greater wisdom by relying on the collective thoughts, ideas, and beliefs of those who surround me.”

Their reflection echoes how emotional intelligence, conflict management, and communication can help them learn from others more broadly across settings. This style of leadership can enable teams to solve problems together and preempt conflict if consensus, empowerment, and relationships are the goals.

Across these change stories, communication skills (like active listening, asking questions, sharing feelings and needs) strengthen one’s emotional intelligence (in the form of knowing one’s own feelings and limitations, learning from others, taking accountability) in service to conflict management (in the form of building consensus and relationships). This combination of behaviors composes one aspect within one’s self-awareness of personal leadership growth as participants take on more equity-centered leadership styles to build consensus and form sustainable partnerships with peers by showing empathy for and learning from them.

#### Visioning organizational change

The additional competencies of leading change/change management, organizational culture, and visioning had high factor loadings in our CFAs across multiple time points. In these stories, participants galvanized teams with a shared vision as part of their process in leading and managing cultural change in their organizations and communities; in part by using the emotionally intelligent communication skills (described in the previous section) to garner contributions from all.

A clinician from Cohort 1 outlined their team’s CS-inspired “blueprint” to intentionally create and sustain a center for LGBT healthcare, which included: crafting a mission, vision, and values statement in addition to working with stakeholders who might or might not share similar values for social justice, creating a brand to promote the center, and writing grants to fund their mission. In addition to these concrete steps, this participant shared how this project impacted them personally, their organization, and the larger community:

“Through all of these steps, I have constantly evaluated my own personal struggles and successes in my new role as an organizational leader of change. We now have a Gender Wellness Center and have fulfilled all of the lofty goals that seemed so elusive three years ago. I have developed new confidence in my ability to lead efforts to sustain this project in the future. The culture of health in our organization around the care of [LGBT] people has improved considerably, thanks to these efforts, and the regional [LGBT] community expresses gratitude and appreciation for the services offered in our center.”

The skills in change management helped this participant bring together people with different values to realize a vision of social justice and instigate change in their organization and region. Likewise, an administrator from Cohort 2 listed the opportunities in which they convened decision-makers to gain others’ endorsement of and contribution to their vision (and their academic department’s mission) for community engagement (CE):

“Though there are several leaders who support this new mission, there are also few who do not support this mission. Their resistance is due to understanding the meaning, value, and rationale of CE at a medical school. Some of the leaders’ resistance was solely due to sharing the financial pie with a new mission. [My task was] to get support from nearly all critical leaders for this mission and its strategic plan [by]:

1. Meeting one on one with many leaders and getting their buy in for this mission and my ideas
2. Visiting many departments and giving presentations on CE
3. Recruiting some key leaders to vocalize their support for CE
4. Establishing a steering committee and bringing [together] voices of a large number of leaders [in] shaping the strategic plan
5. Showing substantial financial and nonfinancial return on investment (ROI) for the institutional investment in CE”

This participant leveraged others’ perspectives to implement a strategic plan (with a unified vision and mission) for an award-winning program as part of an organizational shift toward CE.

In addition to policy and planning, other participants’ roles in cultural change (at their respective institutions) involved influencing organization-wide practices. A practitioner from Cohort 1 wrote about how the poverty simulation activity in the program helped them sympathize with others, as well as see how systemic conditions exacerbate existing challenges that patients living in poverty might face. They explained how they applied their learnings to promote a vision of health equity and create change in their organization’s approach to serving this community:

“Through the Clinical Scholars program, I have honed my leadership skills, including the power to influence people and create partnerships necessary to solve [W]icked [P]roblem[s] of health inequity. Through these skills, I was able to bring the Poverty Simulation to the leadership of [my city’s] Children’s Hospital to increase understanding of the needs of the patients we serve. Currently, the Poverty Simulation is part of the training for incoming nurse residents at the hospital. The leadership skills, such as self-awareness, negotiation, communication, developing partnerships, innovation, advocacy, and strategic thinking have been my catalysts towards advancing health equity, not just within the projects that we are working on as Clinical Scholars but also beyond.”

This participant’s ability to communicate their vision of health equity and build partnerships impacted how their organization changed the way they train practitioners, in service to providing more empathetic care.

In their stories, participants used emotionally intelligent communication skills (highlighted in the previous section) as well as steps in managing organizational change to assemble diverse stakeholders under a shared vision of health equity. This ability to unite others under a compelling vision enabled them to influence cultural change in their organizations that made use of multiple stakeholders’ contributions, rather than hinging on the actions of a single participant. This organizational change, in turn, increased access to health care to underserved populations or improved community engagement.

## Discussion and conclusion

Our qualitative analysis of participants’ most significant change stories show that the competency of self-awareness has multiple facets. First, the combination of communication, emotional intelligence, and conflict management (which were highly correlated with self-awareness in the competency assessment results) can manifest as empathy, learning from others, and consensus building. Participants can also use these interpersonal skills to unite multiple stakeholders, with diverse sets of values, under a shared vision of social justice that can generate cultural change in organizations where all members’ contributions are welcomed and valued. This latter process exemplifies the competencies of visioning, leading change/change management, and organizational culture, respectively (which had high factor loadings in our confirmatory factor analyses). Together, our mixed-methods approach used assessments that were representative of all program participants and in-depth accounts of learning to illustrate broader impacts of individual-level growth.

One limitation of our findings stems from the self-selection into equity-centered leadership programs like Clinical Scholars. Because the cohorts included more white women who are more advanced in their careers (compared to other demographics), these results may not be as applicable to participants in other training programs with broader target audiences.

Participation in Clinical Scholars evaluation also differed across cohorts and data collection methods. While the response rates were comprehensive for quantitative surveys, not all program participants may have had a pertinent qualitative change story to share for myriad reasons. However, our findings show that those who submitted a change story did not differ significantly (in terms of relevant self-assessed competencies) from those who abstained. Our goal with the qualitative data analysis was to illustrate the constructs that were (substantively and statistically) significant to the cohort, as gathered by the representative survey data. Ultimately, we argue that our mixed-method approach is a robust way to analyze representative quantitative data to support qualitative analysis of participants’ complementary accounts at multiple levels or contexts to illustrate a latent construct.

Prior studies [11] demonstrated that the dimension of self-efficacy and competency of self-awareness showed the most change among participants. In our preliminary analyses, there was a dip in self-assessments between Months 6 and 18 (midpoint), which plateaued in Month 36 (endpoint). To further examine self-awareness, we also relied on participants’ descriptions of growth in their change stories to identify skills, abilities, and attitudes that made up their leadership styles. This qualitative analysis allowed us to illustrate the overall impact of the program, despite fluctuations in self-assessed competencies over time.

Meanwhile, our qualitative analysis revealed few themes around impact when focusing on labeling change stories with all 25 competencies assessed in the comprehensive evaluation survey. The quantitative analyses supplemented that initial open coding and narrowed our focus to six competencies that all cohorts perceived growth in, over time. This process allowed us to pinpoint how these competencies were theoretically connected in their operational definitions, as well as empirically linked in the change stories, resulting in a richer illustration of self-awareness of personal leadership growth.

Additionally, we recommend one-on-one or focus group interviews so that moderators can probe for more detail as needed to collect richer change stories. Over time, the cohorts’ accounts shrank in depth, only citing the outcome of career advancement, as a result of their participation in the program, which may have been impacted by increased responsibilities and other constraints throughout the pandemic. Our mixed-method approach, however, allowed us to focus our thematic analysis on more novel combinations of competencies and showcase richer stories across cohorts that also echoed the competencies measured in all five cohorts of participants.

In conclusion, our sequential, explanatory mixed-methods design allowed us to support our hypothesis that a latent construct existed among additional competencies related to self-awareness that were salient across all five cohorts of participants while also expanding on what self-awareness of personal leadership growth consisted of, from their perspectives. This mixed-method approach can extend other evaluation research designs to understand nuanced latent constructs and how individual attitudes and actions connect with broader programmatic impacts at the community- or organizational-level.

## Supporting information

Mckelvy et al. supplementary material Mckelvy et al. supplementary material

## Supplementary material

The supplementary material for this article can be found at https://doi.org/10.1017/cts.2024.597.
