# Supplementary material for: Mixed-methods analysis of personal growth in an equity-centered leadership development program
Source: J Clin Transl Sci. 2024 Nov 8;8(1):e175. doi: 10.1017/cts.2024.597 (PMC11604511; doi:10.1017/cts.2024.597)
Supplement: Mckelvy et al. supplementary material [file S2059866124005971sup001.docx]

# Supplemental Materials

| Supplementary Table 1. Domains, competencies, dimensions of the Clinical Scholars Competency Evaluation Survey, administered at four time points | | | | | | |
| --- | --- | --- | --- | --- | --- | --- |
|  | |  | Time point: Month 0 (Baseline) | | | |
| Competencies by Domain | |  | Self-efficacy (dimension) | Attitude  (dimension) | Knowledge (dimension) | Intent to use (dimension) |
| Personal Leadership (domain) | |  |  |  |  |  |
|  | Commitment to Intercultural Development |  | 1-7 | 1-7 | 1-7 | 1-7 |
|  | Emotional Intelligence ^a^ | (EIN) | 1-7 | 1-7 | 1-7 | 1-7 |
|  | Self-Awareness | (SAW) | 1-7 | 1-7 | 1-7 | 1-7 |
|  | Social Justice |  | 1-7 | 1-7 | 1-7 | 1-7 |
| Interpersonal Skills (domain) | |  |  |  |  |  |
|  | Communication ^a^ | (COM) | 1-7 | 1-7 | 1-7 | 1-7 |
|  | Conflict Management ^a^ | (CMG) | 1-7 | 1-7 | 1-7 | 1-7 |
|  | Innovation Orientation |  | 1-7 | 1-7 | 1-7 | 1-7 |
|  | Negotiation |  | 1-7 | 1-7 | 1-7 | 1-7 |
|  | Practice of Multi-Culturalism |  | 1-7 | 1-7 | 1-7 | 1-7 |
|  | Visioning ^b^ | (VIS) | 1-7 | 1-7 | 1-7 | 1-7 |
| Organizational Impact (domain) | |  |  |  |  |  |
|  | Organizational Capacity for Advancing Health Equity |  | 1-7 | 1-7 | 1-7 | 1-7 |
|  | Organizational Culture ^b^ | (IOC) | 1-7 | 1-7 | 1-7 | 1-7 |
|  | Diversity and Inclusion |  | 1-7 | 1-7 | 1-7 | 1-7 |
|  | Implementation Science/Evidence-Based Practice |  | 1-7 | 1-7 | 1-7 | 1-7 |
|  | Performance Management for Innovation |  | 1-7 | 1-7 | 1-7 | 1-7 |
|  | Leadership Change/Change Management ^b^ | (LCM) | 1-7 | 1-7 | 1-7 | 1-7 |
|  | Systems Thinking |  | 1-7 | 1-7 | 1-7 | 1-7 |
|  | Political Thinking |  | 1-7 | 1-7 | 1-7 | 1-7 |
| Community and Systems Impact (domain) | |  |  |  |  |  |
|  | Advocacy |  | 1-7 | 1-7 | 1-7 | 1-7 |
|  | Collaboration and Partnerships |  | 1-7 | 1-7 | 1-7 | 1-7 |
|  | Futuring |  | 1-7 | 1-7 | 1-7 | 1-7 |
|  | Health Equity |  | 1-7 | 1-7 | 1-7 | 1-7 |
|  | Meaningful Community Engagement |  | 1-7 | 1-7 | 1-7 | 1-7 |
|  | Social Determinants of Health |  | 1-7 | 1-7 | 1-7 | 1-7 |
|  | Stakeholder Analysis |  | 1-7 | 1-7 | 1-7 | 1-7 |
| Note: Each of the 162 participants self-assessed four dimensions for each of the 25 competencies (i.e., 100 items per survey) at four time points, generating 400 possible ratings for each participant. ^a^ Top three competencies with statistically significant correlations with Self-Awareness & large effect sizes across four time points; ^b^ Competencies with the top three loadings per time point in CFA models. | | | | | | |

| Supplementary Table 2. Means and standard deviations for the mean competency scores (related to Self-Awareness) at Month 36 (endpoint), by participants who abstained or submitted a Most Significant Change (MSC) story | | | | | | | | | |
| --- | --- | --- | --- | --- | --- | --- | --- | --- | --- |
|  | |  | Abstained | | Submitted | |  | | |
| Competencies by Domain | |  | *M* | *(SD)* | *M* | *(SD)* | *t* | *df* | *p* |
| Personal Leadership (domain) | |  |  |  |  |  |  |  |  |
|  | Social Justice | (SJU) | 5.83 | (0.88) | 6.03 | (0.70) | -1.37 | 105.40 | 0.17 |
|  | Self-Awareness | (SAW) | 5.96 | (0.70) | 6.11 | (0.55) | -1.33 | 104.02 | 0.19 |
|  | Emotional Intelligence ^a^ | (EIN) | 6.10 | (0.84) | 6.20 | (0.73) | -0.67 | 109.29 | 0.50 |
| Interpersonal Skills (domain) | |  |  |  |  |  |  |  |  |
|  | Conflict Management ^a^ | (CMG) | 5.58 | (1.01) | 5.73 | (0.80) | -0.84 | 104.68 | 0.40 |
|  | Communication ^a^ | (COM) | 5.96 | (0.67) | 6.02 | (0.52) | -0.51 | 104.09 | 0.61 |
|  | Visioning ^b^ | (VIS) | 5.65 | (0.96) | 5.62 | (1.05) | 0.20 | 113.95 | 0.84 |
| Organizational Impact (domain) | |  |  |  |  |  |  |  |  |
|  | Leadership Change/Change Management ^b^ | (LCM) | 5.61 | (0.97) | 5.62 | (0.85) | -0.06 | 109.82 | 0.96 |
|  | Organizational Culture ^b^ | (IOC) | 5.79 | (0.92) | 5.74 | (0.81) | 0.31 | 110.06 | 0.76 |
| Note: n=162 participants. Competency scores range from 1 to 7. ^a^ Top three competencies with statistically significant correlations with Self-Awareness & large effect sizes across four time points; ^b^ Competencies with the top three loadings per time point in CFA models. *p < .05. | | | | | | | | | |

**Prompt for soliciting Most Significant Change (MSC) stories**

We are interested in understanding the impact that you feel your participation in the program has had in your own story.  Please describe in one or two paragraphs the most significant change that has resulted from your involvement with the Clinical Scholars Program.  Think about this like telling a story.  Please describe the situation, task, actions, results, or other details you can relate to the change.  You are welcome to submit more than one story, but [we] would ask that you not provide more than two and that each story would be described in detail, not just a laundry list of concepts or ideas. You are also free to do a video recording if that would be easier for you—simply record yourself telling your story—it can be done simply with a phone video and you sitting in a chair or standing in a quiet room. You will also need to answer the one follow-up question in your video if you chose to use a video. ​

After completing your story, please answer the one question that follows about your story. If you would rather record yourself telling your story rather than writing it, you are free to do so—just don’t forget to answer the follow-up question as the final part of telling your story. ​

Follow up question: Reflecting on the story that you provided, which of the following categories best fits the story: ​

- Personal leadership development and individual growth ​
- Organizational impact on your or another organization ​
- Community impact, where “community” is defined broadly to include a geographic location, a defined group of people, a field of work, etc.​


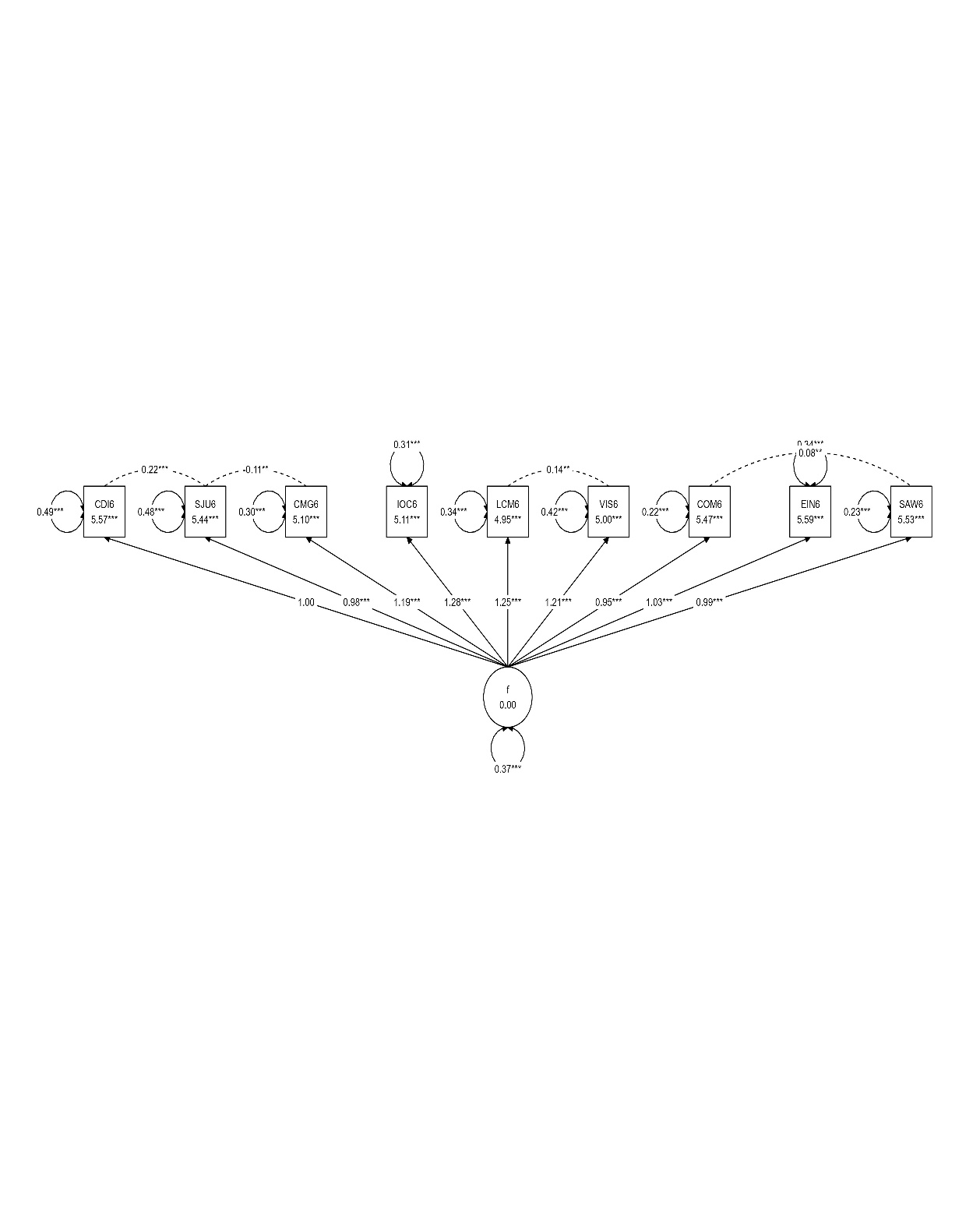


Supplementary Figure 1 Confirmatory factor model for Month 6 with unstandardized loadings


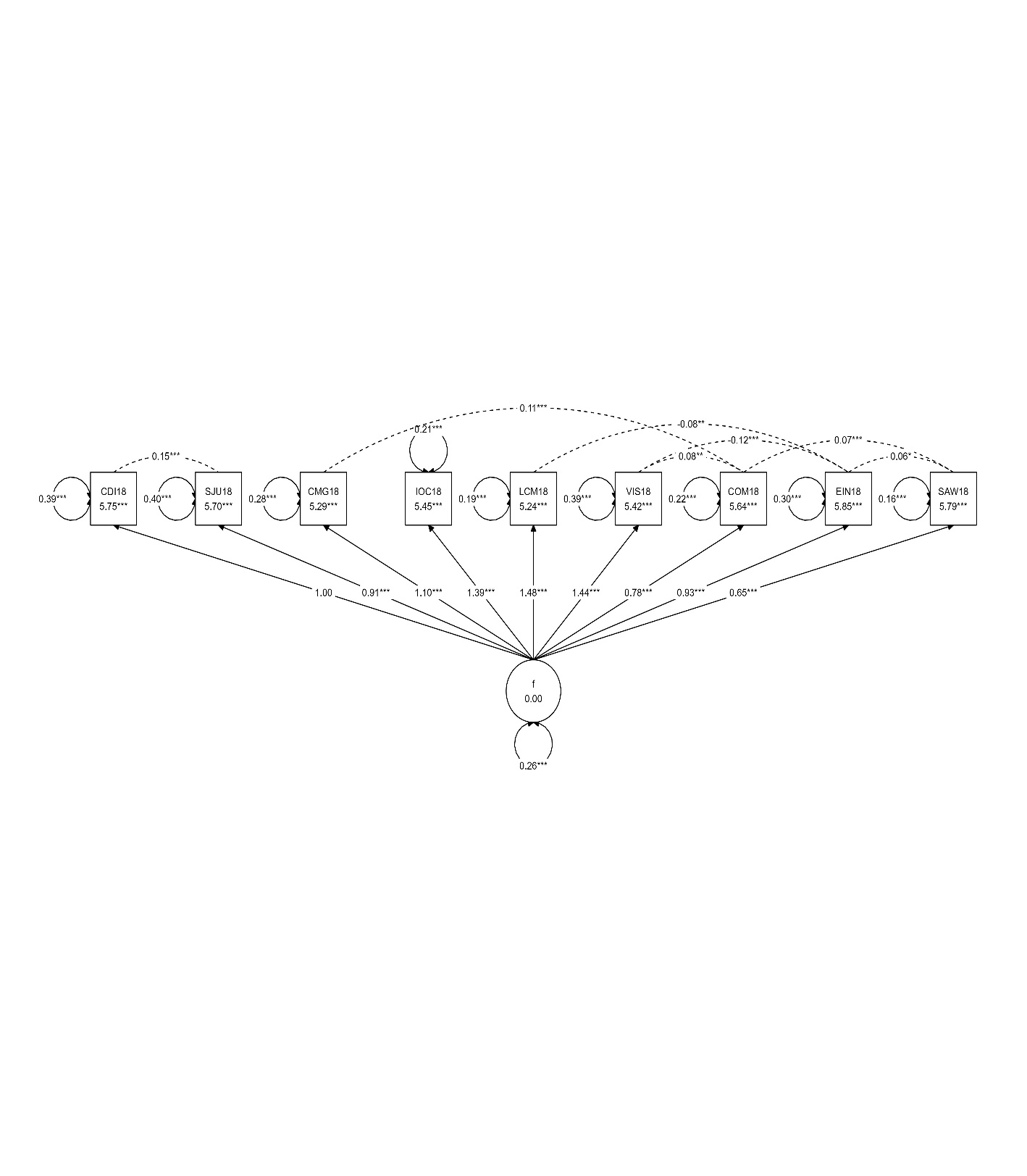


Supplementary Figure 2 Confirmatory factor model for Month 18 with unstandardized loadings


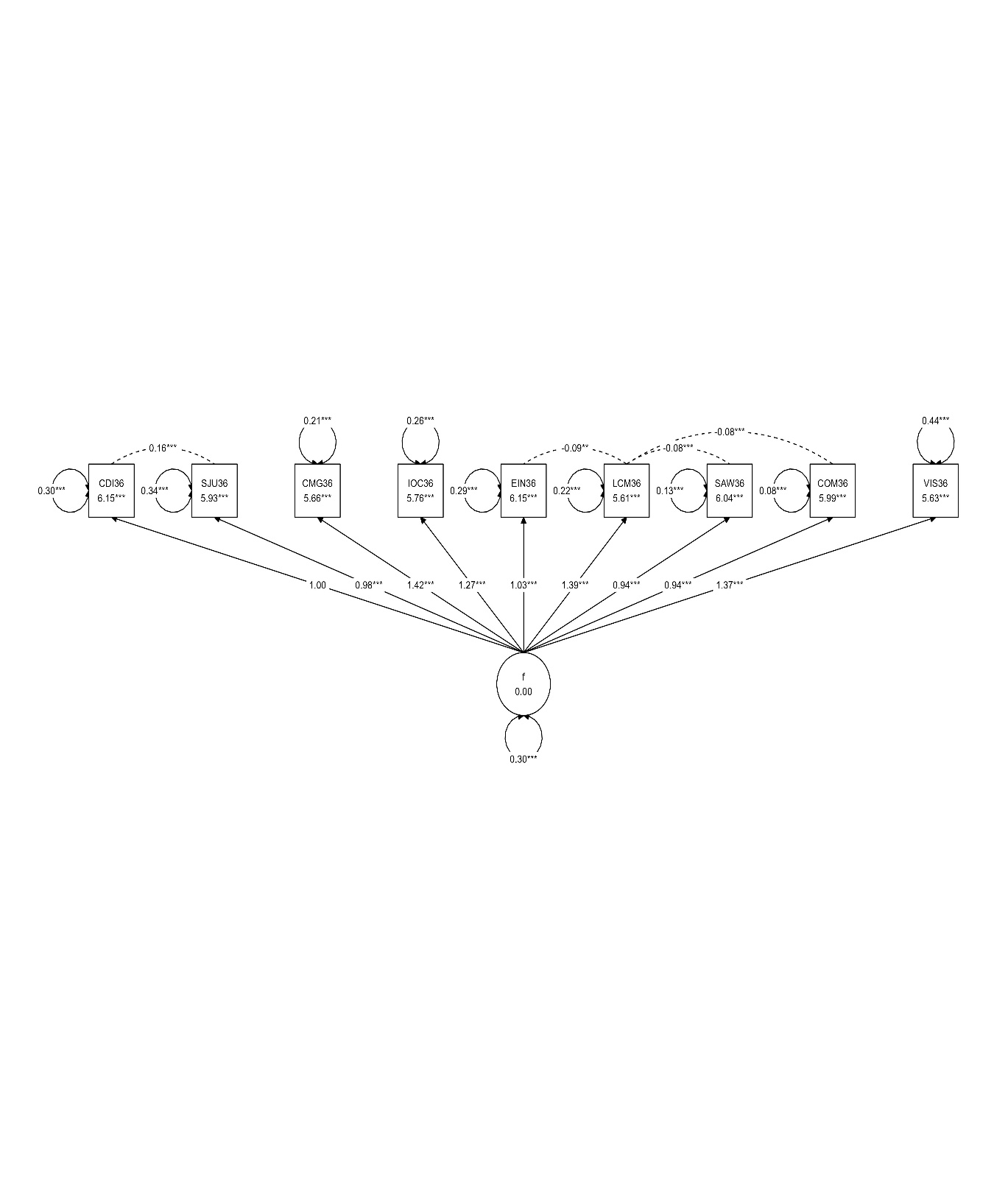


Supplementary Figure 3 Confirmatory factor model for Month 36 with unstandardized loadings
